# Gene expression profiling of normal thyroid tissue from patients with thyroid carcinoma

**DOI:** 10.18632/oncotarget.8820

**Published:** 2016-04-18

**Authors:** Roberto Ria, Vittorio Simeon, Assunta Melaccio, Giovanna Di Meo, Stefania Trino, Carmela Mazzoccoli, Ilaria Saltarella, Aurelia Lamanuzzi, Annalisa Morano, Angela Gurrado, Alessandro Pasculli, Gaetano Lastilla, Pellegrino Musto, Antonia Reale, Franco Dammacco, Angelo Vacca, Mario Testini

**Affiliations:** ^1^ Department of Biomedical Sciences and Human Oncology, Section of Internal Medicine “G. Baccelli”, Bari, BA, Italy; ^2^ Laboratory of Pre-Clinical and Traslational Research, IRCSS CROB, Rionero in Vulture, PZ, Italy; ^3^ Department of Biomedical Sciences and Human Oncology, Unit of Endocrine, Digestive and Emergency Surgery, Bari, BA, Italy; ^4^ Department of Pathology, University of Bari “Aldo Moro” Medical School, Bari, BA, Italy

**Keywords:** gene expression profile, hypoxia, microenvironment, oncogenes, thyroid cancer

## Abstract

Gene expression profiling (GEP) of normal thyroid tissue from 43 patients with thyroid carcinoma, 6 with thyroid adenoma, 42 with multinodular goiter, and 6 with Graves-Basedow disease was carried out with the aim of achieving a better understanding of the genetic mechanisms underlying the role of normal cells surrounding the tumor in the thyroid cancer progression. Unsupervised and supervised analyses were performed to compare samples from neoplastic and non-neoplastic diseases. GEP and subsequent RT-PCR analysis identified 28 differentially expressed genes. Functional assessment revealed that they are involved in tumorigenesis and cancer progression. The distinct GEP is likely to reflect the onset and/or progression of thyroid cancer, its molecular classification, and the identification of new potential prognostic factors, thus allowing to pinpoint selective gene targets with the aim of realizing more precise preoperative diagnostic procedures and novel therapeutic approaches.

**STATEMENT OF SIGNIFICANCE:**

This study is focused on the gene expression profiling analysis followed by RT-PCR of normal thyroid tissues from patients with neoplastic and non-neoplastic thyroid diseases. Twenty-eight genes were found to be differentially expressed in normal cells surrounding the tumor in the thyroid cancer. The genes dysregulated in normal tissue samples from patients with thyroid tumors may represent new molecular markers, useful for their diagnostic, prognostic and possibly therapeutic implications.

## INTRODUCTION

Thyroid carcinoma is the most common endocrine malignancy, accounting for about 1% of all types of human cancer, with a growing incidence rate reported worldwide [1]. Over 95% of thyroid carcinomas (TC) derive from follicular epithelial cells [2, 3]. They have been traditionally classified as well differentiated carcinoma, including papillary carcinoma (PTC, 80%) and follicular carcinoma (FTC, 10-15%), whereas poorly differentiated and anaplastic carcinomas account for 1-2% of thyroid malignancies [2]. In addition, medullary carcinoma (MTC, 3-5%) is a malignancy of parafollicular C cells derived from the neural crest and occurs as sporadic (75%) and hereditary (25%) types [4]. This wide spectrum of thyroid cancer histotypes has been closely linked to the pattern of cumulative genetic and epigenetic alterations, which are correlated with tumor differentiation, metastasis and invasion [5].

Several studies have addressed the biological and diagnostic aspects of gene expression profiling (GEP) of this tumor [6]. A high level of complexity is related to the fact that thyroid tumors consist of neoplastic cells irregularly intermingled with normal (connective tissue and vessels) and reactive (stromal and immune) cells. Quantitative relations between these components may vary among patients and even inside a single tumor [7, 8]. Most microarray studies include tumor fragments containing 80-90% of tumor cells and some authors recommend that investigations be carried out on microdissected cells [8]. However, although this step is useful for a reliable assessment of the neoplastic transformation, it precludes the diagnostic use of microarray. Only when the expression signal is strong enough to be detected in biopsy specimens, for example in case of diffuse neoplastic infiltration, is the microarray-based technology applicable for diagnostic purposes.

In the past decades, the major efforts of cancer research have been focused on the malignant cell itself. This has led to the identification of oncogenes, tumor suppressor genes and their associated signaling pathways that modulate growth, survival and proliferation of tumor cells. Pathophysiological interactions of cancer cells with their microenvironment are highlighted by the disease progression and neovascularization, and are witnessed by autocrine/paracrine circuits that activate multiple signaling pathways and affect the most important aspects of malignant phenotype, i.e., apoptosis/survival, proliferation, invasion, and angiogenesis [9].

Here, we have examined the GEP of normal tissue samples from patients with neoplastic and non-neoplastic thyroid diseases, intraoperatively taken on the basis of macroscopic judgement and confirmed by immunohistochemistry. We asked whether differences can be detected between the GEP of normal thyroid tissue from patients with thyroid carcinoma and the GEP of thyroid samples from normal subjects and patients with thyroiditis. Next, we defined the list of the differentially expressed genes on the basis of different gene selection methods. The overall results indicate that an optimal set of genes can be defined with the aim of differentiating neoplastic from non-neoplastic thyroids.

## RESULTS

### Histological features and validation of samples

**Figure 1:**
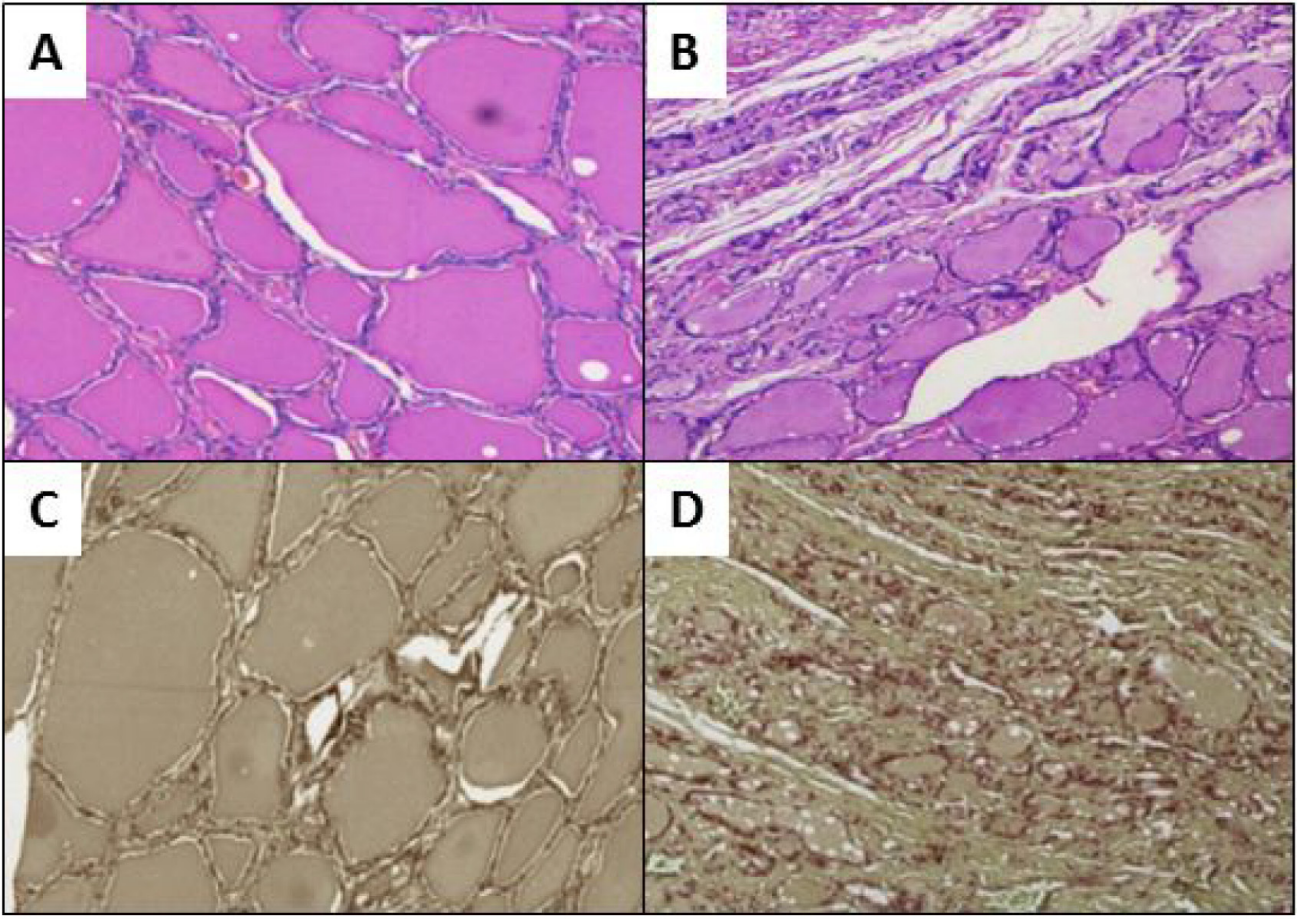
Evaluation of neoplastic infiltration Ematoxylin/eosin staining showing non neoplastic thyroid tissues in panel **A.** versus neoplastic infiltration in panel **B.** Immunohistochemical staining for Cytokeratin 19: in panel **C.** CK19 is not detected in normal tissue, while diffuse immunoreactivity for CK19 in a papillary thyroid cancer infiltration is visible in panel **D.**

Table 1 summarizes the histological diagnoses made in the 97 patients with thyroid diseases included in this study. Histology showed 43 malignancies, 42 benign diseases, 6 hyperfunctioning diseases, and 6 thyroid adenomas. The absence of neoplastic infiltration of samples was established by hematoxylin/eosin standard protocol and subsequent immunohistochemistry on adjacent sections. Of the 97 samples, only four resulted positive for carcinoma micrometastases and were excluded from analysis (Figure 1).

**Table 1:** General characteristics, cytological and diagnostic features of the patients studied

Characteristics	Total number (percentage)
Patients	97 (100)
Sex: M/F	30 (31) – 67 (69)
Age, median (range) years	53.5; range 24 – 85
Pre-operative cytology	50
- TYR 1	6 (12.0)
- TYR 2	6 (12.0)
- TYR 3	24 (48.0)
- TYR 4	2 (4.0)
- TYR 5	12 (24.0)
Thyroid cancer	43 (44.3)
Multinodular goiter	39 (40.2)
Thyroiditis	3 (3.1)
Thyroid adenoma	6 (6.2)
Graves-Basedow disease	6 (6.2)

### Gene expression profiles

**Figure 2:**
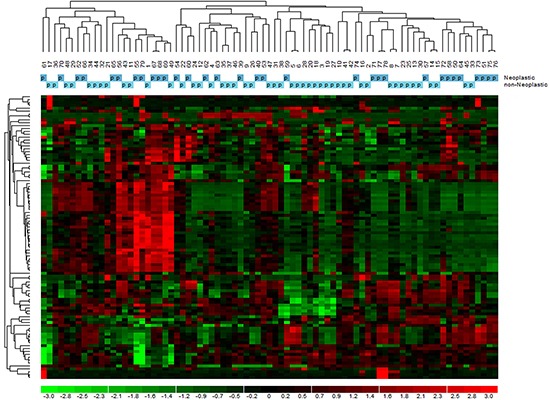
Unsupervised analysis of gene expression profiles in normal tissue of tumoral and non-tumoral thyroids The dendrogram was generated with a hierarchical clustering algorithm based on the average-linkage method. In the matrix, each column represents a sample, and each row a gene.

GEP was carried out on 76 samples. To determine whether normal thyroid tissues derived from neoplastic thyroids could be distinguished from those from non-neoplastic thyroids according to the natural grouping of their GEP, we performed an unsupervised analysis using the hierarchical clustering algorithm on the 1.5 average fold change probes in samples dataset. The probe sets found to be highly variable along the entire data set generated a dendrogram (Figure 2) with two major branches and five secondary branches. Neither normal tissues derived from neoplastic thyroids nor those from non-neoplastic thyroids could be identified as a distinct cluster of the dendrogram. The most significant modulated functions recognized for the probe sets were associated to oncogenesis, cell stress response, together with cell death and growth processes.

**Figure 3:**
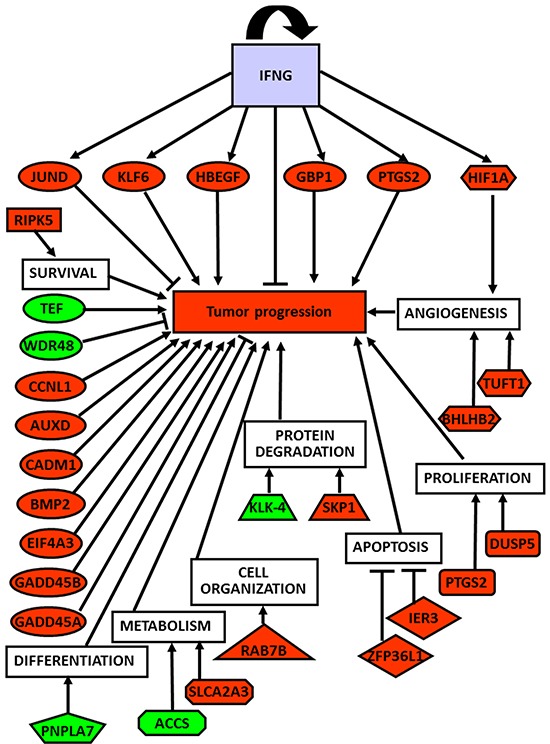
Global role of up- and down-regulated genes in thyroid cancer development and progression Correlation of up- (red-labeled) and down-regulated (green-labeled) studied genes and their corresponding biological functions with tumor progression.

A supervised analysis was performed to find which genes specifically differentiated normal tissues from neoplastic and non-neoplastic thyroids. Forty-five differentiating genes were detected: 35 up-regulated (Supplementary Table S1), and 10 down-regulated (Supplementary Table S2). Interestingly, the differentially expressed genes were involved in tumorigenesis and cancer progression, angiogenesis and response to hypoxia, cell survival, proliferation, apoptosis, cell organization, protein degradation, cell differentiation and metabolism (Figure 3).

### GeneMania gene network analysis

**Figure 4:**
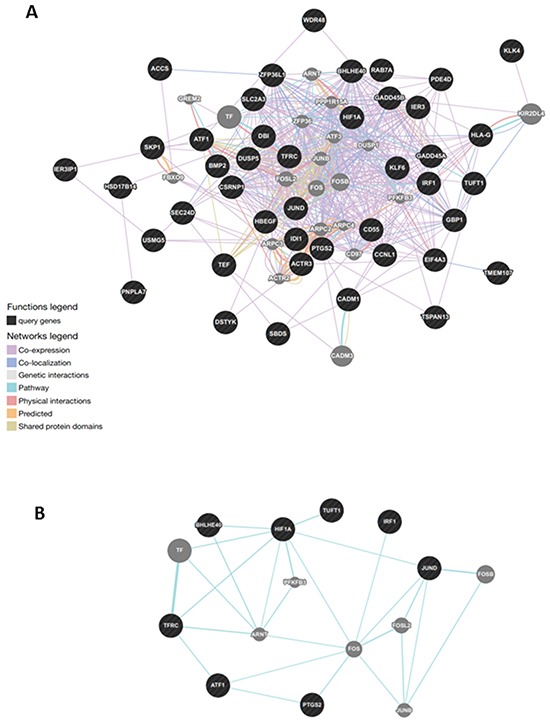
Gene network analysis in normal tissue of tumoral thyroids **A.** General analysis was based on protein-protein interaction and pathway databases with the nucleocytoplasmic transport-related genes (1.29-fold; P,0.05). GeneMANIA retrieved known and predicted interactions between these genes and added extra genes (grey circles) that are strongly connected to query genes (dark circles). **B.** Functional gene network in normal tissue of patients with thyroid cancer. Correlation of the three genes, namely *HIF1A*, *TUFT1*, *BHLHB2*, involved in angiogenesis and response to hypoxya with other disregulated genes, particularly JUND.

This revealed a dense co-expression network (Figure 4, panel A). Two of queried genes (*EIF3CL* and *FAM47E*) did not show any correlation with others. The network included 65 genes with 852 interactions among them. Several genes presented at least 10 connections. Figure 4, panel B reports functional gene networks separated from the general network, which involve 8 out of the 45 genes: *HIF1A*, *TUFT1*, *BHLHB2* (also known as *BHLHE40*), *IRF1*, *JUND*, *PTGS2*, *ATF1*, and *TFRC*.

### Real-time RT-PCR validation

**Figure 5:**
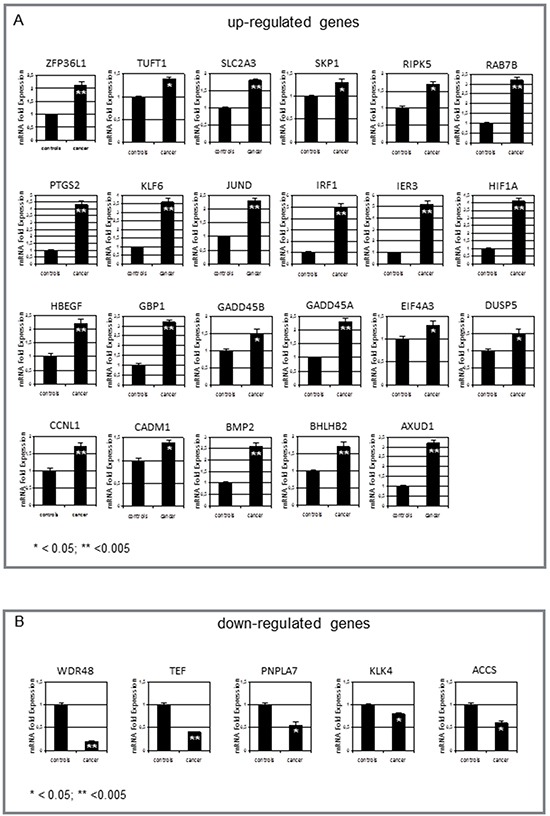
Measurement of gene expression by real-time RT-PCR **A.** Significant increase in normal tissues from neoplastic vs non-neoplastic thyroids of *ZFP36L1*, *TUFT1*, *SLCA2A3*, *SKP1*, *RIPK5*, *RAB7B*, *PTGS2*, *KLF6*, *JUND*, *IRF1*, *IER3*, *HIF1A*, *HBEGF*, *GBP1*, *GADD45A*, *GADD45B*, *EIF4A3*, *DUSP5*, *CCNL1*, *CADM1*, *BMP2*, *BHLHB2*, *AUXD1*. **B.** In contrast, only 5 genes were down-regulated in these tissues: *WDR48*, *TEF*, *PNPLA7*, *ACCS*, and *KLK-4*. Values are expressed as mean ± 1SD for 32 normal tissues from neoplastic thyroids and 28 non-neoplastic thyroids. Significance of changes was calculated by the Wilcoxon-Wilcox test.

The GEP data were validated on 60 samples of normal tissues from neoplastic (32 samples), and non neoplastic thyroids (28 samples) by testing mRNA of the differentially expressed genes. Real-time RT-PCR of the confirmed up- and down-regulated genes is shown in Figure 5. Twenty-three genes, involved in angiogenesis and response to hypoxia, were found to be up-regulated (p<0.05 for all genes) in normal tissues from neoplastic thyroids, whereas 5 genes were down-regulated (p<0.05 for all genes - namely, the tumor suppressor gene *WDR48*, the transcription factors *TEF*, *PNPLA7* and *ACCS* involved in adipocyte differentiation and metabolism, and the kallikrein protease KLK4). However, a differential expression could not be shown for 18 genes by RT-PCR.

### Ingenuity analysis on confirmed genes

**Figure 6:**
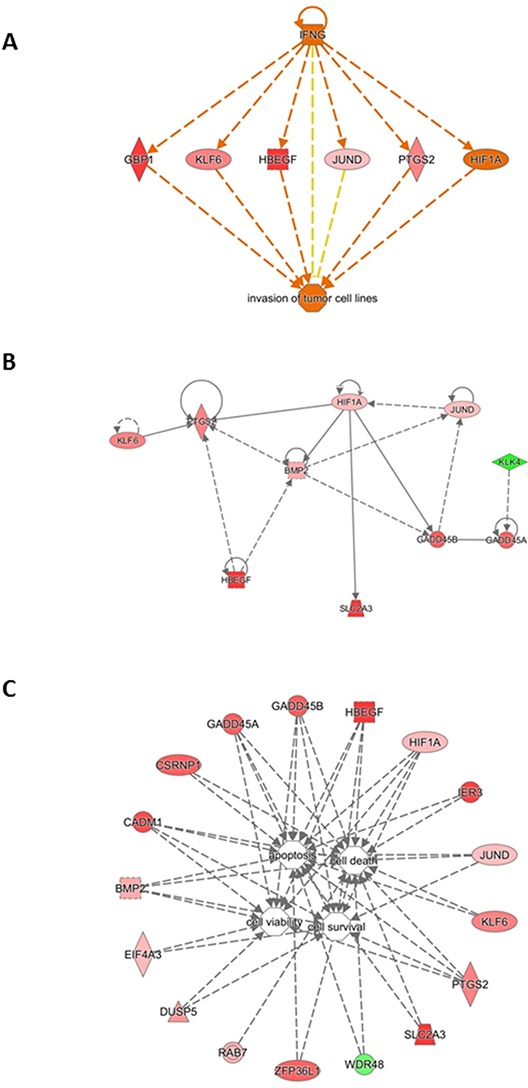
Gene interaction network based on information from the Ingenuity Pathways Knowledge Base under the IFNG control **A.** Correlation with cancer cell invasion. **B.** Gene interaction network based on information from the Ingenuity Pathways Knowledge Base under the HIF1A control. Genes that are up- or down-regulated are labelled in red and green, respectively. **C.** Gene interaction network based on information from the Ingenuity Pathways Knowledge Base related to cell survival. Correlation of genes dysregulated in tumoral thyroids with cell viability, survival, death and apoptosis. Genes that are up- or down-regulated are labelled in red and green, respectively.

We algorithmically generated a gene interaction network based on the connectivity of focused genes. Regulator Effect network, which integrates the upstream regulator results with those of the downstream effects, was used to generate a cause-and-effect hypothesis. The analysis could explain how upstream regulators may cause particular phenotypic and functional outcomes downstream. This network analysis indicated two regulators, namely *IFNG* (Figure 6, panel A) and *HIF1A* (Figure 6, panel B), and a connection of gene pathways for modulation of cell viability and survival as well as cell death and apoptosis (Figure 6, panel C). These interactions could favor thyroid tumor initiation and progression.

## DISCUSSION

Previous GEP studies of thyroid tissues from patients with neoplastic diseases, which were designed to identify genes probably involved in the initiation and progression of thyroid cancer, revealed that thyroid tumor cells express a genomic profile different from that of normal cells [10]. However, findings in solid and hematologic cancers also suggest that modulation of the microenvironment, rather than genetic alterations of the tumor cells *per se*, may partly account for the tumor initiation and progression. Signals from microenvironment are thought to play a critical role in inducing and maintaining tumor cell growth, migration and survival [11, 12]. They stem from reciprocal positive and negative interactions between tumor cells and stromal cells (endothelial cells, fibroblasts, macrophages, mast cells, lymphocytes), and are mediated by an array of cytokines, receptors, and adhesion molecules [13].

Here, the comparative analysis of normal tissues from neoplastic and non-neoplastic thyroids identified 28 differentially expressed genes, which may play an important role in tumor initiation and progression. Specific pathways analysis indicated their involvement in the control of apoptosis, metabolism, cell movement, cell response to hypoxia, and cell proliferation. All these genes are related among them (Figure 4, panel A) and can be suggested as markers of the neoplastic involvement of the thyroid tissue.

The interplay between dysregulated genes is likely to be a *primum movens* of the tumor initiation and progression. In fact, *KLF6*, *JUND*, *HBEGF*, *GBP1*, *GADD45B*, *GADD45A*, *EIF4A3*, *CCNL1*, *CADM1*, *BMP2*, *AUXD1* and the down-regulated *WDR48* (tumor suppressor gene) and *TEF* (transcription factor) are all genes involved in tumorigenesis and tumor progression. They are also linked to the genes governing angiogenesis and response to hypoxia (*HIF1A*, *TUFT1*, *BHLHB2*), cell survival (*RIPK5*), proliferation (*PTGS2*, *DUSP5*), apoptosis (*ZFP36L1*, *IER3*), metabolism (*SLCA2A3*), cell organization (*RAB7B*), protein degradation (*SKP1*, *KLK-4*), and adipocyte differentiation and metabolism (*PNPLA7*, *ACCS*) (Figure 3, panel A).

Worth of note is the correlation with the three genes: *HIF1A*, *TUFT1*, and *BHLHB2* that were up-regulated in normal tissues from neoplastic thyroids. These genes are involved in angiogenesis and response to hypoxia and control other dysregulated genes, particularly *JUND* (Figure 4, panel B).

Hypoxia is a major angiogenic stimulus, and hypoxia-inducible factor-1 (*HIF-1*) is the master regulator of the cellular response to hypoxia [14]. In several human tumors [15] *HIF-1α* overexpression is positively related to growth, angiogenesis [16], chemoresistance [17], and poor prognosis [18]. Under normoxia, reactive oxygen species (ROS) can activate *HIF-1α*, thus stimulating its transcriptional activity [17, 19, 20]. Finally, some oncogenes are able to induce *HIF-1α* overexpression [14]. Here, in normal tissues of neoplastic thyroids the oncogene *JUND* was overexpressed and directly correlated with the *HIF-1α* overexpression (Figure 5, panel A). The *HIF-1α* overexpression can be responsible for the activation of *PTGS2* and *KLF6*, *HBEGF*, *BMP2*, *SLC2A3* and *GADD45A*/ *B* (Figure 5, panel A). These activated pathways are related to response of cells to hypoxia as well as other stresses, and induce cell survival and proliferation. As evidenced in Figure 6, panel A, all these genes are also under the control of cytokines such as IFN-gamma, that mediates cancer progression and drug resistance [20].

*RIPK5* is a dual Ser/Thr and Tyr kinase [21] which integrates both extracellular stress signals transmitted by various cell-surface receptors and signals derived from intracellular stress. It represents a crucial regulator of cell survival and death. *IER3*, a member of the “immediate early response gene” family, is another stress-inducible gene with anti-apoptotic activity that plays a pivotal role in cell survival under stress conditions such as hypoxia [22, 23].

Among up-regulated genes that could play a role in cell viability and survival, we found *ZFP36L1*, a zinc finger protein that regulates various cellular processes by binding to adenine uridine rich elements in the 3′ untranslated regions of sets of target mRNAs to promote their degradation. In lymphoid malignancies, *ZFP36L1* interacts with and mediates degradation of, BCL2 mRNA through which it regulates its pro-apoptotic effects (Figure 6, panel C) [24].

Two genes *SLCA2A3*, that are important for glucose transport, and *RAB7B*, a small GTPase that regulates transport between the different compartments of the endomembrane system in eukaryotic cells, have been found to be up-regulated in normal tissues from neoplastic thyroid patients. These genes increase access to glucose to support the high rate of glycolysis and satisfy the great need of energy in tumor cells [25]. *RAB7B* also controls the transport between late endosomes and the trans Golgi network, interacts directly with myosin II, regulates actin remodeling and, consequently, influences cell adhesion, polarization and migration [25].

Lastly, S-phase kinase associated protein 1 (*SKP1*) serves as an adaptor to bind the F-box protein in the SCF (Skp1/Cul1/F-box protein) complex. SCF mediates degradation processes in G1 phase and the response to mitogen stimulation in tumor cells [26].

Taken together, our results show that the dysregulated (up- or down-regulated) genes and their pathways detected in the normal tissue distant from the neoplastic tissue are fundamental for cell response to various stresses as well as for cancer development, survival and progression (Figure 3). Whether these changes are influenced by the tumor microenvironment and/or tumor cells or are associated with genomic alterations in normal cells is now being investigated in our laboratory. It is conceivable that microenvironmental factors (such as hypoxia, inflammation, expression of multiple cytokines and growth factors, etc.) regulating tumor-associated stromal elements may display unstable, heterogeneous and progressive characteristics to an extent comparable with (and causally linked to) the instability of the cancer cell genome. In addition, those factors may have genetic causes and consequences (i.e., increased expression of oncogenes, loss of tumor suppressor genes) [27]. This reciprocal interrelationship and heterogeneity may translate into site- and stage-specific changes in the regulation of normal cells from neoplastic thyroids, eventually leading to changes in the proliferation and anti-apoptotic potential of tumor cells, even in the same patient.

Finally, the 28 genes confirmed as differentially expressed in normal tissues from neoplastic vs non-neoplastic thyroids may represent new molecular markers for prognostic stratification of patients and predictors of the possibility to develop cancer [28]. Furthermore, the GEP of thyroid normal tissues, an example of which is shown in this study, may lead to the identification of new therapeutic targets [29], including dysregulated genes, for the management of thyroid cancer patients. In fact, several studies [9, 30, 31] have been focused on novel drugs targeting both cancer cells and the microenvironmental cells. Promising results have been obtained so far, but most cancers still remain incurable malignancies, indicating that the role of microenvironment is important in cancer progression, although its role is still incompletely defined.

Overall, our findings imply that normal cells from neoplastic thyroids: *i)* are functionally different from those from inflammatory or normal thyroids, *ii)* are characterized by an active phenotype, *iii)* resemble transformed cells because they down- or up-regulate some genes like tumor cells, *iv)* may represent a predictive indicator of neoplastic disease even when imaging and/or FNAB are negative or doubtful.

## PATIENTS AND METHODS

### Patients

A triple-blind prospective study was performed between March 2013 and October 2014 on 97 consecutive patients undergoing thyroid surgery, whose demographic, diagnostic and cytological features are summarized in Table 1. The study was approved by the local Ethical Committee of the University of Bari Medical School, and all patients gave their informed consent in accordance with the Declaration of Helsinki.

All patients were submitted to preoperative workup, including measurement of thyroid function and autoantibodies, serum calcium, phosphorus, and magnesium. Ultrasound color doppler imaging for thyroid volume determination, and chest and neck radiography were also performed. Thyroid scintigraphy and fine-needle aspiration cytology were limited to patients with hyperthyroidism (11.0%) and ultrasound-detected nodules (52.0%), respectively.

### Tissue sampling, storage and validation

After patient's consent, a macroscopically normal sample of the gland was taken during operation on the side of the gland opposite to that containing the tumor, or in a macroscopically healthy area when a preoperative diagnosis of malignancy was not known. Samples were stored at −80°C until use.

The absence of neoplastic infiltration of samples was evaluated on adjacent 6 μm 4% paraformaldehyde-fixed paraffin-embedded sections after hematoxylin/eosin standard staining, followed by immunohistochemistry with a specific primary antibody [32, 33].

### Isolation of RNA and microarray analysis

With the exception of 17 samples which were excluded because of poor RNA quality, total RNA was extracted from frozen thyroid tissues with TRIzol reagent (Invitrogen, Carlsbad, CA), the concentration determined on a Nanodrop spectrophotometer (Nano-Drop, Wilmington, DE), and quality assessed with the Agilent RNA 6000 Nano Kit on an Agilent 2100 Bioanalyzer (Agilent Technologies, Milan, Italy). For each sample, 300 ng of total RNA was reverse transcribed to synthesize cDNA and biotinylated cRNA according to the Illumina TotalPrep RNA amplification protocol (Ambion; category n. IL1791). Hybridization of 750 ng of cRNA on Illumina HumanHT12 v4.0 Expression BeadChip array (Illumina Inc.), staining and scanning were performed according to the standard protocol (Illumina Inc.). BeadChip was dried and scanned with an Illumina HiScanSQ system (Illumina Inc.).

The intensity files were loaded into the Illumina Genome Studio software for quality control and gene expression analysis. Quantile normalization algorithm was applied on the data set to correct systematic errors: values below a detection score of 0.05 were filtered out and missing values were imputed. Microarray data (raw and normalized) were submitted to Array Express under accession number E-MTAB-3796.

### Gene ontology analysis

Unsupervised analyses were applied to a subset of genes whose average expression varied at least 1.5 fold from the mean across the whole panel. For hierarchical agglomerative clustering, Pearson's correlation coefficient and average linkage [34] were respectively used as distance and linkage methods in DNA-Chip Analyzer (dChip) software [35].

Differently expressed genes (DEGs) were selected with differential score (DiffScore) cutoff set at ±13 (p<0.05). The DEGs list included 45 genes, and was used to evaluate the functional behavior in terms of Biological Processes performing an enrichment analysis with Ingenuity Pathway Analysis (IPA) - (Ingenuity Systems, Redwood City, CA; http://www.ingenuity.com).

### GeneMANIA analysis

GeneMANIA (version 3.2.1, http://www.genemania.org/) analysis of the DEGs was performed on normal tissues from neoplastic and non-neoplastic thyroids. It finds genes related to a set of input genes, using a very large set of functional interaction data. We analysed the gene network to identify gene–gene interactions, the topology of this gene correlation, and putative additional genes that may be involved in normal tissues from neoplastic and non-neoplastic thyroids if they are shown to interact with a large number of genes in the query set. The association data of GeneMANIA algorithm was selected from the pathway and the protein-protein interaction databases.

### Real-time RT-PCR validation of microarray data

This was performed as described [36]. One μg of total RNA was reverse transcribed into total cDNA with the “iScript cDNA Synthesis Kit” (Bio-Rad). Primers (Supplementary Table S3), together with a fluorochrome FAM- or VIC-labeled TaqMan probe, were premixed at the optimal concentration for amplification. Reaction mixture and amplification conditions were done according to the manufacturer's instructions (Applied Biosystems). Each RNA was tested in triplicate and the threshold cycles values averaged ± 1 SD. The relative gene expression (fold change) in normal thyroid tissue from neoplastic and non-neoplastic thyroids was measured with the comparative threshold cycle (Ct) method using glyceraldehyde-3-phosphate dehydrogenase as endogenous control and the 2–ΔΔCt formula [37].

### Interaction network analysis by IPA for RT-PCR validated genes

The differently validated genes were further analyzed using the IPA software. This all-in-one web-based software, which makes use of the Ingenuity Pathways Knowledge Base (IPKB), generates interaction networks of focus genes based on manually curated information from the literature. The underlying algorithm maximizes connectivity, leading to networks that are likely to represent significant biological function. Briefly, a file containing gene identifiers (ID), their corresponding fold change and p-values were uploaded. Homo sapiens as the species and thyroid as the tissue were also specified. Enrichment of the focus genes in the networks (which always consist of 28 genes) were assessed via Fisher's exact test and used to rank the networks. Furthermore, the software identifies top functions and diseases associated with each network via enrichment scores, highlighting the biological significance of the results.

## SUPPLEMENTARY TABLES


