# Supplementary material for: Gene expression profiling of normal thyroid tissue from patients with thyroid carcinoma
Source: Oncotarget. 2016 Apr 18;7(20):29677–88. doi: 10.18632/oncotarget.8820 (PMC5045425; doi:10.18632/oncotarget.8820)
Supplement: Supplementary file 1 [file oncotarget-07-29677-s001.pdf]

SUPPLEMENTARY TABLES

Supplementary Table S1: Supervised analysis: upregulated genes, relative protein and function

GENE	Log_2 FOLD CHANGE	PROTEIN	FUNCTION
**ZFP36L1**	1.158833	ZFP36 ring finger protein-like 1	Post-transcriptional expression of targeted mRNAs
**TUFT1**	0.752711	Tuftelin 1	Adaptation to hypoxia
**TSPAN13**	0.410653	Tetraspanin 13	Cell development, activation, growth and motility
**TMEM107**	0,601506	Transmembrane protein 107	Ciliogenesis and Sonic hedgehog signaling
**TFRC**	0,579422	Transferrin receptor	Iron metabolism
**SLC2A3**	1.494932	SLC2A3 solute carrier family 2 member 3	Glucose metabolism
**SKP1**	0.413275	S-phase kinase-associated protein 1	Ubiquitination of specific protein for degradation by proteosome
**SEC24D**	0.408036	SEC24 family member D	Involved in vesicle trafficking
**SBDS**	0.508901	Shwachman-Bodian-Diamond syndrome	RNA metabolism
**RIPK5**	0.454913	Receptor interacting protein kinase 5	Cellular organization
**RAB7B**	0.553007	Member RAS oncogene family	Coordinator of cytoskeletal organization
**PTGS2**	0.933491	PTGS2 prostaglandin-endoperoxidesynthase 2	Prostanoid biosynthesis involved in inflammation and mitogenesis
**KLF6**	0.936957	Kruppel-like factor 6	Involved in human carcinogenesis
**JUND**	0.490391	DNA-binding protein, components of the activator protein-1 (AP-1) transcription factor	Tumor angiogenesis, cell differentiation, proliferation, and apoptosis
**IRF1**	1.10503	Interferon regulatory factor 1	Antiviral, antitumor and immune regulatory protein
**IER3IP1**	0.344041	Immediate early response 3 interacting protein 1	Cell differentiation and apoptosis
**IER3**	1.410131	Immediate early response gene 3	Apoptosis
**IDI1**	0.388407	Isopentenyl-diphosphate delta isomerase 1	Apoptosis
**HLA-G**	0.567199	Major histocompatibility complex, class I, G	Immune response
**HIF1A**	0.524236	Hypoxia inducible factor 1, alpha subunit	Adaptation to hypoxia
**HBEGF**	1.506648	Heparin-binding EGF-like growth factor	Cancer cell growth and invasion
**GBP1**	1.475773	Guanylate binding protein 1, interferon-inducible	Drug resistance
**GADD45B**	1.21735	Growth arrest and DNA-damage-inducible, beta	Genomic stability
**GADD45A**	1.192829	Growth arrest and DNA-damage-inducible, alpha	Genomic stability
**EIF4A3**	0.516152	Eukaryotic translation initiation factor 4A3	Translation initiation, nuclear and mitochondrial splicing, and ribosome and spliceosome assembly
**DUSP5**	0.784491	Dual specificity phosphatase 5	Cell proliferation and differentiation

(*Continued*)

GENE	Log_2 FOLD CHANGE	PROTEIN	FUNCTION
**DBI**	0.424356	diazepam binding inhibitor	Lipid metabolism
**CD55**	0.398866	CD55 molecule	Regulation of the complement cascade
**CCNL1**	0.733585	Cyclin L1	Involved in several types of cancer
**CADM1**	1.348437	Cell adhesion molecule 1	Involved in oncogenesis and spermatogenesis
**BMP2**	0.622228	Bone morphogenetic protein 2	Bone and cartilage formation
**BHLHB2**	1.329005	Basic helix-loop-helix transcription factor 2	Development, cell differentiation, cell growth, cell death, oncogenesis, immune systems, circadian rhythm, and homeostasis
**AXUD1**	1.189885	Cysteine- Serine-Rich Nuclear Proteins (CSRNP)-1	Nuclear proteins that contain cysteine- and serine-rich domains
**ATF1**	0.292736	Activating transcription factor 1	Growth, survival, and other cellular activities
**ACTR3**	0.649174	ARP3 actin-related protein 3 homolog	Cell shape and motility through lamellipodial actin assembly and protrusion

Supplementary Table S2: Supervised analysis: downregulated genes, relative protein and function

GENE	Log_2 FOLD CHANGE	PROTEIN	FUNCTION
**WDR48**	-0.42019	WD repeat domain 48	Suppressors of tumor cell survival
**USMG5**	-0.43412	Up-regulated during skeletal muscle growth 5 homolog	Cellular energy metabolism
**TEF**	-0.62444	Thyrotroph embryonic factor	Transcription factor
**PNPLA7**	-0.57649	Patatin-like phospholipase domain containing 7	Adipocyte differentiation
**PDE4D**	-0.6606	Phosphodiesterase 4D, cAMP-specific	Proliferation-promoting factor
**KLK4**	-0.63853	Kallikrein-related peptidase 4	Proteases
**HSD17B14**	-0.45747	Hydroxysteroid (17-beta) dehydrogenase 14	Steroids metabolism
**FAM47E**	-0.36974	Family with sequence similarity 47, member E	Gene expression
**EIF3CL**	-0.43021	Eukaryotic translation initiation factor 3, subunit C-like	Involved in translation
**ACCS**	-0.5252	Acetyl-coenzyme A carboxylase	Fatty acid metabolism

Supplementary Table S3: Primers for RT-PCR

GENE	PRIMERS	
	FORWARD	REVERSE
**ZFP36L1**	*5’- GATGACCACCACCCTCGT-3’*	5’-CTGGGAGCACTATAGTTGAGCA-3’
**WDR48**	5’- TGGGACAATTCGCCTTTGGTC-3’	5’-TGTCAGGGTTTCTTAGGTCTGT-3’
**TUFT1**	5’ -TGGACCCTAGCATGAGT-3’	5’-CGTTCTTGATCCGAAGC-3’
**TSPAN13**	5’-TGTTCCAAGAACTGCCTGTG-3’	5’-AACAAGAAGATGCCCACTGC-3’
**TEF**	5’-ACCATCTTCCTCTACTGCCATCTTTCAG-3’	5’-GTACTTGGTCTCGTACTTGGACACGATG-3’
**SLC2A3**	5’-TTCGTCTCTAGCCTGCACTG-3’	5’-ACACAACTTCTCCGGGTGAC-3’
**SKP1**	5’-GTCTCCTTAACACCGA-3’	5’-CACAACATTTCACTTCTC-3’
**SEC24D**	5′-CTCGAGATGCAGGTTCCATCTGGATATGG-3′	5′-GCGGCCGCTACTTACAAATCTGCTGCTG-3′
**RAB7B**	5’-AGAGAGAATTCATGAATCC CCGGAAGAAGGT-3’	5’-AGAGAGACGTCGACTCTGCTCC TTGACTGGTCTG-3’
**PNPLA7**	5’-GCCTCTGTACCTGCCCTGCT-3’	5’-CTGTATGCAGGGCTGCTGGT-3’
**PDE4D**	5’- TGGATGAGCAGGTGGAAGAG-3’	5’- CACAAACGAAAGGCATGGAA-3’
**KLK4**	5’- GGCACTGGTCATGGAAAACGA-3’	5’- TCAAGACTGTGCAGGCCCAGCC-3’
**KLF6**	5’- CTCTCAGCCTGGAAGCTTTTAGCCTAC-3’	5’- ACAGCTCCGAGGAACTTTCTCCCA-3’
**JUND**	5’-TGTCCTGCCCAGTGTTTGTA-3’	5’-GAGGTTGGGGGCTACTTTTC-3’
**IRF1**	5’-AAAAGGAGCCAGATCCCAAGA-3’	5’-CATCCGGTACACTCGCACAG-3’
**IER3IP1**	5’-CAGGGAATTGGTGGATTTGG-3’	5’-TGGCACTCTCATCACGGTTCT-3’
**IER3**	5’-GCCACCCGACATGACATCC-3’	5’-CTGGTGCGCGAGCGTATCC-3’
**HIF1A**	5′-TTTACCATGCCCCAGATTCAG-3′	5′-GGTGAACTTTGTCTAGTGCTTCCA-3′
**HBEGF**	5′-TCCTCCAAGCCACAAGCACT-3′	5′-AGAAGCCCCACGATGACCAG-3′
**GBP1**	5′-AAGAGAGGACCCTCGCTCTTA-3′	5′-ATGCCTTGGTTAGGGGTGAC-3′
**GADD45B**	5′-GCTGGCCATAGACGAAGAAG-3′	5′-GCCTGATACCCTGACGATGT-3′
**GADD45A**	5′-GCTACTGGAGAACGACAAGAG-3′	5′-CCATTGTGATGAATGTGGGTTC-3′
**DUSP5**	5′-GTGCTGAACTAGGGGAGCTG-3′	5′-AGATGGTGGGTGAACAGGAG-3′
**CCNL1**	5′-TAGGCGGAGTCGATCTGGAA-3′	5′-CCATGGTGCTTGCTTTTATGG-3′
**CADM1**	5′-TTTTCTAGCAGTGAACTCAAAGTATCAT-3′	5′-GATATCGATCATCAGATTACGTGGTG-3′
**BMP2**	5′-ATAGCAGTTTCCATCACCGAA-3′	5′-ACTTCCACCACGAATCCAT-3′
**BHLHB2**	5′-ACTCTTCTGTCTTGCTCCAAGC-3′	5′-AGCAGGATCCCCTAGAGAGTTT-3′
**AXUD1**	5′-TCCGCCGCCGTTTAAAG-3′	5′-ATCGATGGCATCCGCTGT-3′
**ATF1**	5′-TCCGCCGCCGTTTAAAG-3′	5′-ATCGATGGCATCCGCTGT-3′
**ACTR3**	5′-GGGGAATTCACCATGGCGGGACGGC TGCCGGCCTGTGTGGTG-3′	5′-CAGCTCCTCGCCCTTGCTCACCAT GCTCCCGCCGCCGCTGGACATGACTCC AAACACTGG ATTGTGACG-3′
**GAPDH**	5′-CCCTCCAAAATCAAGTGGGG-3′	5′-CGCCACAGTTTCCCGGAGGG-3′
